# Pathogenetic Mechanisms of Hepatitis C Virus-Induced B-Cell Lymphomagenesis

**DOI:** 10.1155/2012/807351

**Published:** 2012-07-11

**Authors:** Fabio Forghieri, Mario Luppi, Patrizia Barozzi, Rossana Maffei, Leonardo Potenza, Franco Narni, Roberto Marasca

**Affiliations:** Section of Hematology, Department of Oncology, Hematology, and Respiratory Diseases, University of Modena and Reggio Emilia, 41100 Modena, Italy

## Abstract

Hepatitis C virus (HCV) infection is probably the most common chronic viral infection and affects an estimated 180 million people worldwide, accounting for 3% of the global population. Although the liver is considered to be the primary target, extrahepatic manifestations are well recognized among patients with chronic HCV infection. Epidemiological studies have clearly demonstrated a correlation between chronic HCV infection and occurrence of B-cell non-Hodgkin's lymphomas (B-NHL). The clinical evidence that antiviral therapy has a significant role in the treatment at least of some HCV-associated lymphoproliferative disorders, especially indolent B-NHL, further supports the existence of an etiopathogenetic link. However, the mechanisms exploited by HCV to induce B-cell lymphoproliferation have so far not completely clarified. It is conceivable that different biological mechanisms, namely, chronic antigen stimulation, high-affinity interaction between HCV-E2 protein and its cellular receptors, direct HCV infection of B-cells, and “hit and run” transforming events, may be combined themselves and cooperate in a multifactorial model of HCV-associated lymphomagenesis.

## 1. Introduction

Hepatitis C virus (HCV) is an enveloped positive, single-stranded RNA virus, belonging to the Flaviviridae family [1]. During its replicative cycle it goes through a negative-stranded RNA, but not DNA, intermediate, so that integration of HCV nucleic acid sequences into the host genome seems unlikely. The HCV genome encodes a single polyprotein precursor of approximately 3000 amino acids, which is proteolytically processed by viral and cellular proteases to produce structural (nucleocapsid, E1, and E2) and nonstructural (NS) proteins (NS2, NS3, NS4A, NS4B, NS5A, and NS5B). The HCV envelope proteins consist of two heavily glycosylated proteins, E1 and E2, which act as the ligands for cellular receptors [1, 2].

Human CD81 is the first identified necessary receptor for HCV cell entry, which can directly bind with HCV E2 protein [3, 4]. CD81 is a widely distributed cell-surface tetraspanin that participates in different molecular complexes on various cell types, including hepatocytes, B-lymphocytes, T-lymphocytes, and natural killer cells. It has been proposed that HCV exploits CD81 not only to invade hepatocytes but also to modulate the host immune responses [5].

Infection with HCV affects an estimated 180 million people, accounting for 3% of the global population [6, 7]. HCV is a well-recognized etiologic agent of chronic hepatitis. Although the natural history of HCV infection is highly variable, an estimated 15% to 30% of patients in whom chronic infection develops have progression to cirrhosis over the ensuing three decades, and these latter patients warrant surveillance for complications, including hepatocellular carcinoma (HCC), which develops in 1%–3% of such patients per year [6, 7]. Indeed, the risk of HCC in the HCV-infected population is 23–35 times higher than in noninfected healthy individuals [8, 9].

Although the liver is considered to be the primary target of HCV infection, extrahepatic manifestations, such as mixed cryoglobulinemia (MC), which is a systemic immune complex-mediated disorder characterized by B-cell proliferation that may evolve into overt B-cell non-Hodgkin's lymphoma (B-NHL) in about 10%–20% of patients several years after diagnosis, are often recognized among patients with chronic HCV infection [10–12]. Moreover, epidemiological evidences strongly suggest a close link between chronic HCV infection and *de novo* B-NHL, not complicating the course of MC [13–16]. The possible pathogenetic mechanisms of HCV-induced B-cell lymphomagenesis are reviewed.

## 2. Epidemiologic Association of HCV and B-NHL

Evans and Mueller proposed that either epidemiologic or virologic guidelines need to be fulfilled to support an etiologic role for a virus in a given human cancer [17]. Suggested epidemiologic guidelines included the following: (a) the geographic distribution of viral infection should coincide with that of the tumor; (b) the presence of viral markers should be higher in case subjects than in matched control subjects; (c) viral markers should precede the tumor, with a higher incidence of tumors in persons with the marker than in those without; (d) prevention of viral infection should decrease tumor incidence [17]. Suggested virologic guidelines included the following: (a) the virus should be able to transform human cells in vitro; (b) the viral genome should be demonstrated in tumor cells and not in normal cells; (c) the virus should be able to induce the tumor in an experimental animal [17].

As far as the association between HCV infection and occurrence of B-NHL is concerned, most of the epidemiologic guidelines for causality from Evans and Mueller are met. HCV is associated with certain B-NHL types, especially in geographic areas with HCV endemicity, like Italy, Japan, and Egypt, where prevalence rates range from 20% to 40% [14, 15, 18–21], whereas in nonendemic areas, as Northern Europe, North America and United Kingdom, the prevalence of HCV infection in B-NHL is far less than 5% [22–24]. The possibility is raised that in these latter geographic areas where HCV prevalence among subjects not affected with B-NHL is low, the spread of the virus may be recent, thus not allowing the full consequences on B-NHL development to be observed. Moreover, studies from areas with low HCV prevalence may not have included sufficient numbers of patients to detect a significant association between HCV and B-NHL [16]. Taken together, the epidemiologic analyses demonstrated that the prevalence of HCV infection in patients with B-NHL is approximately 15% [25]. The prevalence of anti-HCV antibodies and/or HCV RNA sequences is significantly higher in patients with B-NHL than in patients with other lymphoid malignancies or in age matched healthy subjects. Furthermore, HCV infection often precedes by years the occurrence of lymphomas [26]. In a recent meta-analysis focusing on 15 studies, the pooled relative risk (RR) of all B-NHL among HCV-positive persons was found to be 2.5 (95% confidence interval (CI), 2.1–3.1) in case-control studies and 2.0 (95% CI, 1.8–2.2) in cohort studies [27]. Another meta-analysis reviewed data from 23 studies (4,049 NHL patients and 1,813,480 controls) and found a stronger association (odds ratio 5.70) [28]. It should be noted that RR, although moderate (2-3 on average) in comparison to HCV and HCC association, were similarly increased for all major B-NHL subtypes and primary sites of presentation [16, 29]. Only slightly higher RR for extranodal compared with nodal B-NHL were reported for HCV-positive patients, but this difference was largely due to the early studies. Moreover, extensive studies did not demonstrate clear differences on the association between HCV and major histologic B-NHL subtypes, either indolent, namely, follicular, marginal zone (MZL), lymphoplasmacytic, and chronic lymphocytic leukemia/small lymphocytic lymphoma, or aggressive diffuse large B-cell (DLBCL) and Burkitt lymphoma [16, 29, 30]. In fact, earliest studies suggesting a stronger association of HCV with certain subtypes, such as lymphoplasmacytic/Waldenstrom lymphomas, were performed mainly in HCV-infected subjects with MC, a subset of patients in which these lymphoma subtypes have been reported to be highly prevalent [16, 29]. Conversely, one of the largest case-control studies to date found a higher OR (3.5 versus 2.3) for aggressive versus indolent lymphomas, respectively, and suggested that previous data may have also been influenced by the relatively poorer prognosis associated with aggressive lymphomas [14]. Patients with HCV-related DLBCL may have more aggressive clinical features at presentation in comparison to HCV-negative patients [31, 32].

The possible association between specific viral genotypes and malignant lymphoproliferative disorders remains a controversial issue. There are at least six major HCV genotypes whose prevalence varies geographically. Genotype 1 accounts for the majority of infections in North America, South America, and Europe [7]. Various clinical studies failed to demonstrate a link between specific viral genotypes and B-NHL, but it should also be noted that this issue was not specifically addressed in several other series. Luppi et al. documented an unexpectedly lower prevalence of HCV genotype 1b/II in patients with B-NHL. Conversely, the prevalence of genotypes 2a/III and 2b/IV was higher in patients with B-NHL than in either hemodialysis or chronic liver disease patients, thus suggesting that different HCV variants may show greater lymphotropism [33]. Recent epidemiologic evidence from a multicenter retrospective study also suggested that genotype 2 may be more prevalent and carcinogenic in lymphoma patients [34]. In details, HCV-positive patients were classified as cancer patients (129 patients, including 53 hematologic malignancies and 76 solid tumors), immunocompetent (333 subjects) and HCV-HIV coinfected (102 patients). Genotype 1 predominated (84%) in immunocompetent as compared to patients with HCC (74%, *P* = .08) or lymphoma (59%, *P* = .001). By contrast, genotype 2 was more prevalent in patients with lymphoma (24%), compared to immunocompetent (8%, *P* = .003), yielding a 3-fold increase in cancer risk among HCV-infected patients than other genotypes [34]. Interestingly, Pellicelli et al. [19] observed that DLBCL patients had a higher prevalence of genotype 1 and a shorter duration of HCV infection, as compared to patients with indolent, low-grade B-NHL, who showed a higher prevalence of genotype 2 and longer duration of HCV infection. Because HCV genotype 2 is associated with a longer duration of viral infection, it has been speculated that over time it may induce a persistent chronic immunostimulation of B-cells. On the contrary, direct lymphocyte transformation could be hypothesized for HCV genotype 1 in aggressive lymphomas, on the basis of the shorter duration of viral exposure [19]. Future perspective studies enrolling a large number of patients are warranted to further investigate the different distribution and carcinogenic potential of different HCV genotypes.

The regression of HCV-related B-NHL following antiviral therapy probably represents the strongest argument in favor of an etiologic link between HCV infection and certain human lymphomas [16, 26]. Several clinical trials showed that antiviral therapy, mostly based on peg-interferon and ribavirin, resulted in either complete or partial remissions of lymphoma in HCV-positive but not HCV-negative B-NHL patients [29, 35–37]. A systematic review has shown that complete responses were achieved in 75% of the HCV-positive cases [38]. Lymphoma regression was usually positively correlated with viral load reduction [29]. These trials have been conducted in asymptomatic indolent lymphomas during a phase in which no other therapeutic intervention was administered. For aggressive lymphoma or symptomatic indolent lymphoma, HCV eradication alone is not an option. These patients require systemic therapy with rituximab and chemotherapy-based regimens as first treatment. Nevertheless, antiviral therapy to eradicate HCV may be an option after successful lymphoma therapy. Whether HCV eradication after-chemoimmunotherapy may impact future survival outcome remains uncertain [29]. Regarding this topic, La Mura et al. retrospectively analyzed 343 patients affected with NHL [39]. Twenty-five of the 69 HCV-positive subjects received antiviral therapy (interferon and ribavirin) following antineoplastic treatment, in order to eradicate HCV infection. Overall survival (OS) was slightly better in HCV-infected NHL patients treated with antiviral therapy compared with untreated, even if without statistically significance. Conversely, disease-free survival (DFS) was significantly improved in treated versus untreated patients. A sustained virologic response was obtained in 8/25 (32%) HCV-positive NHL patients who underwent antiviral treatment. None of the patients who eradicated HCV infection had a lymphoma relapse at followup, whereas 5/17 of those who did not respond to antiviral therapy experienced relapses. At multivariate analysis, the independent factors related to a better DFS in this series were antiviral therapy and indolent histology at the onset of lymphoma [39]. Antiviral treatment may be a strategy to reinforce the results of successful chemoimmunotherapy regimens, but future prospective studies are needed to further investigate this clinical issue. Of interest, a recent study has shown that HCV-infected patients who had received interferon therapy and had experienced a sustained virologic response had a hazard ratio of lymphomagenesis that was significantly lower than patients who had not received antiviral treatment [40]. These data suggest that antiviral treatment may also be efficacious in preventing lymphomagenesis in HCV-infected patients. Moreover, it should be of interest to investigate the impact of newer directly acting antiviral agents, such as protease inhibitors telaprevir and boceprevir [11, 41–43], on the future prevalence and clinical outcome of B-NHL in patients with chronic HCV infection. While reactivation risk of hepatitis B virus (HBV) after chemoimmunotherapy is well recognized and prophylactic antiviral therapy to suppress HBV-DNA is widely recommended, the issue of HCV reactivation in lymphoma patients undergoing antineoplastic treatments is lesser understood [29, 44]. However, a significant proportion of patients with HCV-positive NHL, when treated with conventional chemoimmunotherapy, may develop liver toxicity due to either direct cytotoxicity or increased drug toxicity from suboptimal drug metabolism [29, 45]. The addition of rituximab to chemotherapy does not seem to impact significantly on liver toxicity [45]. HCV-RNA levels appear to increase during chemoimmunotherapy as a result of viral reactivation, but HCV-RNA levels subsequently decrease at 6 months posttreatment, often without major clinical consequences to most patients [44]. Nevertheless, it should also be noted that massive liver necrosis may occur in HCV-positive lymphoma patients on withdrawal of chemotherapy or reduction of corticosteroids, suggesting an immune-mediated mechanism of hepatic damage [44, 46]. Without initial liver dysfunction, HCV-positive patients with NHL could experience a similar outcome compared with their HCV-negative counterparts, when treated with conventional chemoimmunotherapy [44, 47]. A protective role of antiviral prophylaxis to suppress HCV replication during antineoplastic treatments has not yet been defined [29, 44]. Prospective studies and longer followups are necessary to ascertain whether HCV-positive B-NHL patients have inferior outcome or whether there would be long term consequences of chemoimmunotherapy on the progression of liver disease [47]. Patients with HCV infection and lymphoma are recommended to be carefully monitored for hepatotoxicity and HCV-RNA levels. Furthermore, hematologists and hepatologists should work closely together in order to optimize the management of HCV infection throughout lymphoma treatment and improve clinical outcome [29].

## 3. Mechanisms of HCV-Induced Lymphoproliferation

The biological rational for investigating a causal association between HCV infection and the occurrence of B-NHL is based on epidemiological and clinical observations. Nevertheless, limited information are so far available about the biological mechanisms of HCV-induced lymphoproliferation. Evidences from experimental studies suggest that several different mechanisms may be involved in HCV-mediated B-cell transformation [16, 29, 48].

**Figure 1:**
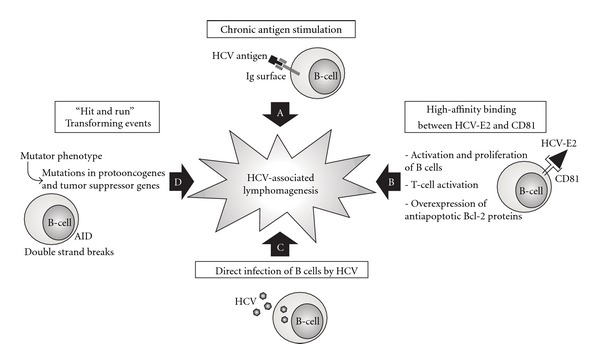
(A–D) The different oncogenetic mechanisms are not mutually exclusive, but they may be integrated and cooperate in a multifactorial pathogenetic model of HCV-associated B-cell lymphoproliferation.

Similarly to the association of *Helicobacter pylori *infection and gastric MALT lymphoma, the concept of chronic antigen stimulation leading to a monoclonal malignant proliferation may also be applied to HCV (Figure 1(A)) [49, 50]. Interestingly, HCV-associated B-NHL generally originate from germinal center (GC) or post-GC B-cells, suggesting that lymphomagenesis occurs when B-cells experience somatic hypermutation and proliferate in response to an antigen [51, 52]. Further evidence comes from the antibody response and immunoglobulin variable (Ig VH) gene usage in patients with chronic HCV infection and HCV-associated B-NHL. In three out of five HCV-positive nodal MZLs, Marasca et al. revealed the usage of the *VH1-69 *gene with similar CDR3, indicating a highly biased and nonrandom use of the *VH *segments in this subtype of tumors [53]. These data indicated the role of a common antigenic epitope involved in the selection and in the expansion of the B-cell clone at the origin of neoplastic cells. The *VH1-69 *immunoglobulin segment is expressed in the restricted repertoire of fetal liver B lymphocytes and is thought to be involved in natural immunity. A productive *VH1-69 *rearrangement is present in 1.6% of normal B lymphocytes in adults. *VH1-69 *is rearranged in 10% to 20% of B-cell chronic lymphocytic leukemia and a *VH1-69 *monoclonal rearrangement is also present in the majority of patients with type II MC, a typical HCV-related disorder [53]. Further experimental sequencing of clonal Ig variable regions from both MC and HCV-associated B-NHL patients documented restricted IgV gene repertoire, with expression of VH and VL genes (VH1-69 and V*κ*3-A27), suggesting exposure and response to a common antigen [54–56]. Of note, HCV-E2 protein is the primary target of antibody responses against HCV [57]. Quinn et al. obtained the cloning of the B-cell receptor from one HCV-positive DLBCL and its expression as a soluble immunoglobulin [58]. The immunoglobulin rescued was shown to bind the HCV-E2 glycoprotein in a manner identical to a bona fide human anti-E2 antibody, suggesting that some HCV-associated B-NHL may originate from B-cells that were initially activated by HCV-E2 protein [58]. Similarly, in a reported case of an HCV-associated plasma cell leukemia, immunoblotting showed that the monoclonal IgG-kappa detected in the serum was directed against a viral protein, namely, the HCV core protein [59]. These and other studies suggest an indirect, antigen-driven lymphomagenetic role of HCV, with HCV-E2 protein recognized as one of the most important antigens involved in chronic B-lymphocyte stimulation [16, 26, 29].

A second mechanism, potentially involved in HCV-associated lymphomagenesis, derives from the high-affinity binding between HCV-E2 and one of its receptors, the tetraspanin CD81, expressed on B-cells (Figure 1(B)) [16]. CD81 is known to form B-cell costimulatory complex with CD19, CD21, and interferon-inducible Leu-13 (CD225) proteins. This complex reduces the threshold for B-cell activation via the B-cell receptor by bridging antigen specific recognition and CD21-mediated complement recognition [60, 61]. It was reported that engagement of CD81 on human B-cells by a combination of HCV E2 protein and anti-CD81 mAb leads to the proliferation of naive B-cells, and E2-CD81 interaction induces protein tyrosine phosphorylation and hypermutation of the immunoglobulin genes in B-cell lines [62]. In addition to direct effects on B-cells, engagement of CD81 on T-cells lowered the threshold for interleukin-2 production, resulting in strongly increased T-cell proliferation. This could lead to T-cell activation in response to suboptimal stimuli and bystander activation of B-cells [63]. Taken together, these results suggest that CD81 engagement on B- and T-cells may lead to direct or indirect activation [16]. Chronic B-cell proliferation, in response to antigenic stimulation or polyclonal activation, may predispose to genetic lesions such as translocation and/or overexpression of the antiapoptotic protein Bcl-2 [64]. In a recent study, human Burkitt's lymphoma cell line (Raji cells) and primary human B lymphocytes (PHB) were treated with HCV-E2 protein and HCV particles produced by cell culture (HCVcc) [65]. The results showed that both E2 and HCVcc triggered phosphorylation of IkB*α*, with subsequent increased expression of NF-kB and NF-kB target genes, such as antiapoptotic Bcl-2 family proteins (Bcl-2 and Bcl-xL). Either Raji cells or PHB cells were protected from FAS-mediated death. In addition, both E2 protein and HCVcc increased the expression of costimulatory molecules CD80, CD86, and CD81 itself, and decreased the expression of complement receptor CD21. The effects were dependent on E2-CD81 interaction on the cell surface, since CD81-silenced Raji cells did not respond to both treatments. Moreover, an E2 mutant that loses the CD81 binding activity could not trigger the responses of both Raji cells and PHB cells. Of note, the effects were not associated with HCV replication in cells [65]. Hence, E2-CD81 engagement plays a role in activating B-cells, protecting B-cells from activation-induced cell death, and regulating immunological function. These latter mechanisms may contribute to the pathogenesis of HCV-associated B-cell lymphoproliferative disorders [65]. Moreover, the interaction between HCV-E2 and CD81 on B-cells has been shown to stimulate the enhanced expression of activation-induced cytidine deaminase (AID) and to induce double-strand DNA breaks in the IgVH gene locus, thereby contributing to a mutator phenotype that increases the risk of B-cell malignant transformation [66].

Another oncogenetic mechanism that has been proposed is the direct infection of B-cells by HCV (Figure 1(C)) [16, 29, 67]. In the early 1990s, the presence of HCV-RNA was demonstrated by PCR not only in serum/plasma and liver tissues but also in peripheral blood mononuclear cells (PBMCs), especially in B-cells, of patients infected with HCV [68–71]. Nevertheless, although HCV has been detected in lymphocytes from HCV infected patients and patients with MC, only in a minority of cases RNA-negative strands, the HCV replicative intermediates suggestive of viral replication, were also detected in the cells [72–74]. Detection of negative-strands HCV-RNA in PBMCs by polymerase chain reaction, may also be due to either contamination or passive absorption of circulating HCV, thus potentially leading to false positive results [75, 76]. Marukian et al. showed that culture-grown HCV replicated in hepatoma cells, but no HCV replication was detected in B- or T-cells, monocytes, macrophages, or dendritic cells from healthy donors [77]. Furthermore, Stamataki et al. have provided experimental evidence that HCV might infect B-cells, but B-cells were not able to support active viral replication [78]. Overall, these results should indicate that PBMC may not be permissive to HCV replication [16].

However, it has been reported that HCV may infect and replicate only in a relatively rare subset of B-cells, such as CD5+ B-cells. These cells have been shown to express high levels of CD81 and to expand in HCV-infected liver [79]. Alternatively, B-cells may need another event, such as coinfection with another virus, namely, Epstein-Barr virus (EBV), to become permissive for HCV infection and replication [16, 80]. Neither HCV-RNA nor viral proteins have generally been detected in lymphomatous cells in vivo, with a few exceptions, for example a primary DLBCL of the liver, found to harbor viral nucleic acids by in situ hybridization and a mantle cell lymphoma case, from which a lymphoma cell line could be established in vitro [26, 81, 82]. Moreover, Sung et al. established a B-cell line (SB) from an HCV-infected B-NHL, whose virions could infect primary human hepatocytes, PBMCs, and an established B-cell line (Raji cells) in vitro [83]. Further studies provided evidence that HCV replicates in various hematopoietic cell types, including peripheral dendritic cells, monocytes, and macrophages [84–86]. Overall, despite the evidence that HCV can infect B-cells, the results about its capacity to replicate in B-cells and other blood mononuclear cells and to induce direct malignant lymphoproliferation still appear highly conflicting [16, 29].

A Japanese group recently established HCV transgenic mice that expressed the full HCV genome in B-cells (RzCD19Cre mice) [87]. Interestingly, RzCD19Cre mice with substantially elevated serum-soluble interleukin-2 receptor **α**-subunit (sIL-2R**α**) levels developed B-NHL. Another mouse model of lymphoproliferative disorder was established by persistent expression of HCV structural proteins through disruption of interferon regulatory factor-1 (*irf-1* ^−/−^/CN2 mice). *Irf-1* ^−/−^/CN2 mice showed extremely high incidences of lymphomas and lymphoproliferative disorders. Moreover, these mice showed increased levels of interleukin (IL)-2 and IL-10, as well as increased Bcl-2 expression, which promoted oncogenic transformation of lymphocytes. In this mouse model, the overexpression of apoptosis-related proteins and/or aberrant cytokine production were the primary events involved in inducing lymphoproliferation [87].

A recent study found that peripheral blood B-cells from patients with chronic HCV infection were infected and also had enhanced gene expression associated with B-NHL development when compared to healthy controls [88]. Furthermore, HCV has been found to induce high mutation frequency of cellular genes (immunoglobulin heavy chain, Bcl-6, p53 and beta-catenin genes), in B-cell lines and PBMCs in vitro, by inducing double strand breaks and by activating error-prone-polymerases and AID [89]. These mutations of cellular genes are amplified in HCV-associated B-NHL in vivo, suggesting that HCV-induced mutations in proto-oncogenes and tumor suppressor genes may lead to oncogenetic transformation of the infected B-cells. The so-called mutator phenotype induced by HCV acute and chronic infection in B-cells may be considered a “hit and run” mechanism of cell transformation (Figure 1(D)) [89].

It has been proposed that HCV uses B-cells as reservoirs for persistent infection, which could result in the enhanced expression of lymphomagenesis-related genes, particularly AID, which is thought to be crucial for the initiation and progression of B-NHL [67]. Other studies suggested that the evolution from lymphoproliferation to malignancy may require a second transforming event such as the antiapoptotic Bcl-2 rearrangement. The t(14;18) translocation is indeed significantly associated with chronic HCV infection and particularly with MC [90, 91]. Although the role of virus penetration and replication in B-cells has still to be fully clarified, several evidences suggest that the presence of HCV virus or HCV proteins in these cells represents an oncogenic stimulus [16, 29].

## 4. Conclusion

Epidemiological studies have clearly demonstrated a correlation between chronic HCV infection and occurrence of B-NHL. The clinical evidence that antiviral therapy has a significant role in the treatment and prevention of some HCV-associated lymphoproliferative disorders, especially indolent B-NHL, further supports the existence of an etiopathogenetic link. The mechanisms exploited by HCV to induce B-cell lymphoproliferation differ from the classical mechanisms of herpesviral-induced lymphomagenesis, which require the maintenance of either EBV or human herpesvirus-8 genomes in the transformed B-cells as clonal episomes, together with the expression of an array of latent and, to a lesser extent, of lytic proteins [92]. It is conceivable that the different mechanisms proposed, namely, chronic antigen stimulation, high-affinity interaction between HCV-E2 protein, and its cellular receptors, direct HCV infection of B-cells and “hit and run” transforming events, are not mutually exclusive, but they may be combined themselves in a multifactorial model of HCV-associated lymphomagenesis (Figure 1(A–D)) [16, 26, 29, 67].
